# High Electrochemical Performance from Oxygen Functional Groups Containing Porous Activated Carbon Electrode of Supercapacitors

**DOI:** 10.3390/ma11122455

**Published:** 2018-12-04

**Authors:** Wen Yang, Yanjie Li, Yanyan Feng

**Affiliations:** 1 Guangxi Key Laboratory of Electrochemical and Magnetochemical Functional Materials, Department of Chemistry and Bioengineering, Guilin University of Technology, Guilin 541004, China; yangwen167@163.com (W.Y.); lyj624731930@163.com (Y.L.); 2 Department of Chemical Engineering, Sichuan University, Chengdu 610065, China

**Keywords:** oxygen-containing groups, carbon electrode, supercapacitor, high electrochemical performance

## Abstract

Carbon electrode materials for double layer capacitors have attracted much attention, due to their low cost and abundant sources. Their low specific capacitance, however, hinders the development of carbon electrode materials. In this paper, the large specific surface area commercial activated carbons, rich in micropores, were initially oxygen-functionalized by treatment using concentrated H_2_SO_4_, saturated (NH_4_)_2_S_2_O_8_, and H_2_SO_4_/(NH_4_)_2_S_2_O_4_ mixed oxidants, respectively. The as-prepared samples were analyzed using N_2_ adsorption/desorption isotherms, X-ray photoelectron spectroscopy, and Boehm titration, and used as electrode materials for supercapacitors. Characterization results displayed that the oxidation treatment decreased the specific surface area along with increasing oxygen content. The electrode test showed that the electrochemical activity increased as oxygen content increased. The result that oxygen-functionalized activated carbon, even with a lower specific surface area but much more oxygen content, had higher capacity than pristine activated carbon, tells of the critical role of oxygen functional groups. The excellent capacitive performance suggests a good potential for oxygen functional carbon material to be a highly promising electrode material for supercapacitors.

## 1. Introduction

Inexpensive, stable, and much efficient energy storage devices are very crucial for the growing demand for sustainable energy and electric vehicles. Supercapacitors (SCs) have been extensively acknowledged as backup power devices, energy conservation, and power tools, and are expanding their applications in electrical vehicles [1,2,3,4]. A broad variety of appropriate electrode composites have been explored, mainly built upon charge accommodation at the electric double layer and the occurrence of Faradaic reactions [5]. These electrode materials are mainly constituted by metal oxides, conductive polymers, and porous carbon materials. Of the extensively studied electrode materials of transition metal oxides, RuO_2_ displayed the excellent initial electrochemical performance, whereas the cost of precious metal oxides and difficulties in large-scale production limited its practical applications [6,7]. Likewise, conductive polymers possessed lots of shortcomings, e.g., poor stability. Therefore, porous carbon materials have emerged as the most potential alternative, owing to their cheapness, high supply, outstanding conductivity, eco-friendliness, and steady structural characteristics.

Carbon electrode materials for double layer capacitors have attracted much attention, due to their low cost and abundant resources. The low specific capacitance, however, hindered the development of carbon electrode materials. Recently, the main interest has been focused on tailoring pore structure, specific surface, graphitization, and morphology of carbon materials [5,8,9]. As is well known, porous carbon suffered from the serious drawback of low capacity. The surface availability is low for the generation of electric double layers due to great difficulty in ions diffusion within most porous carbon electrode materials [10], hence, attempts to improve the capacitive behavior by increasing the specific surface area of carbon materials are often restricted. Therefore, it is crucial to take into account a new strategy that does not depend upon the ion charge accommodation at the electric double layer. Within our work, we successfully developed an inexpensive, porous, activated carbon material, rich in oxygen-containing groups, and studied the effect of oxygen functional groups upon the capacitive performance; a redox reaction built upon oxygen-functionalized carbon electrode materials was proposed, to explain the much improved capacitive behavior.

Herein, we examined the influence of treatment strategies on the physiochemical properties of all samples. Textural properties were analyzed using N_2_ adsorption/desorption isotherms. The surface chemistry property was characterized using X-ray photoelectron spectroscopy (XPS) and Boehm titration. Finally, the electrochemical tests results showed that the enhanced capacitive performance was evident consequence of pseudocapacitance, due to introduction of oxygen functional groups.

## 2. Materials and Methods

### 2.1. Sample Preparation

The large specific surface area coconut shell-activated carbons (ACs) (purchased from Songshan Filter Activated Carbon Factory, Gongyi, Henan, China), rich in micropores, was initially oxygen-functionalized by treatment, separately, using concentrated H_2_SO_4_ (98 wt %) (Xilong Chemical Co. Ltd., Guangzhou, China), saturated (NH_4_)_2_S_2_O_8_ (Xilong Chemical Co. Ltd., Guangzhou, China) and H_2_SO_4_/(NH_4_)_2_S_2_O_4_ mixed oxidants. Typically, 10 g of the commercial ACs was placed in 200 mL of concentrated oxidants at 25 °C for 24 h. The proportion of H_2_SO_4_/(NH_4_)_2_S_2_O_4_ was 5.6:100 (v/v) in the mixed oxidants. After the treatment, the resulting materials were washed off using the distilled H_2_O until the filtrate was neutral, then dried at 110 °C for 24 h, and finally stored in dry machine (Shanghai Jing Hong Laboratory Instrument Co. Ltd., Shanghai, China) before use. All samples were referred to as AC-o, AC-s, AC-a, and AC-m, where o, s, a, and m represented the pristine sample, or H_2_SO_4_, (NH_4_)_2_S_2_O_4_, and the mixed oxidants, respectively.

### 2.2. Characterization

Textural characterization of the samples was carried out using N_2_ adsorption/desorption at 77 K with a NOVA1000e surface area and pore size analyzer (Quantachrome Instruments, Florida, USA). Prior to the measurements, samples were degassed at 393 K for 3 h. The specific surface area was calculated from the N_2_ adsorption isotherm by applying the Brunauer-Emmett-Teller (BET) equation, the total pore volume was determined at P/P_0_ of 0.986, and the pore size distribution was calculated using a Density Functional Theory (DFT) model.

The morphology of the samples was conducted by field emission scanning electron microscopy (FESEM, Hitachi SU5000, Tokyo, Japan).

X-ray photoelectron spectroscopy (XPS) measurements were carried out to investigate the surface oxidation state of the samples. The spectra were recorded on an XSAM800 spectrometer equipped with an EA-125 hemispherical multichannel electronics (Kratos Analytical Ltd, Manchester, UK) analyzer operating at a constant pass energy. The samples were loaded on the holder using a carbon adhesive tape. The background pressure in the analysis chamber was maintained below 10^−9^ Pa during the test. The radiation used was the Al Kα line, and the X-ray source was run at a power of 180 W. The C 1s peak position was set at 284.6 eV, and taken as an internal standard.

Boehm titration was used to measure the quantities of oxygen-containing groups over the AC surface. In this method, different oxygen groups can be distinguished by their neutralization behaviors according to the following assumptions: (1) NaOH (Xilong Chemical Co. Ltd., Guangzhou, China) (0.25 M) can neutralize all acid oxygen functional groups; (2) NaOH (Xilong Chemical Co. Ltd., Guangzhou, China) (0.05 M) can neutralize carboxyl, phenol, and lactone groups; (3) Na_2_CO_3_ (Xilong Chemical Co. Ltd., Guangzhou, China) (0.05 M) can neutralize carboxyl and lactone groups; and (4) NaHCO_3_ (Xilong Chemical Co. Ltd., Guangzhou, China) (0.05 M) can neutralize only carboxyl groups. The amount of each base neutralized by oxygen functional groups can be determined by back titration using HCl (Xilong Chemical Co. Ltd., Guangzhou, China) (0.05 M).

### 2.3. Electrochemical Measurements

The working electrode was prepared by mixing the AC with polyvinylidene fluoride (PVDF) (Changshu Xinhua Chemical Co. Ltd., Suzhou, China) and carbon black (Jiaozuo Hexing Chemical Industry Co. Ltd., Jiaozuo, China) (8.5:1:0.5) in *N*-methyl 2-pyrrolidone (NMP) (Chengdu Kelong Chemical Reagent Factory, Chengdu, China) to form a homogeneous slurry. The slurry was coated on a nickel foam (Kunshan Longshengbao Electronic Materials Co. Ltd., Suzhou, China) with a surface area of 1 cm^2^. The electrodes were dried at 105 °C for 12 h, pressurized under 10 MPa, and then weighted. The mass loading of the active material was in the range of 3.0~4.0 mg/cm^2^.

The capacitive performance of all carbon samples was investigated by CHI660D electrochemical (ChenHua Instruments Co., Shanghai, China) working station in 6 M KOH (Xilong Chemical Co. Ltd., Guangzhou, China) using a three-electrode testing cell. In the three-electrode system, graphite electrode and Hg/HgO electrode were applied as the counter and reference electrodes, respectively. The cyclic voltammetry (CV) was performed to study the surface redox reaction on oxygen-functionalized activated carbon (AC) electrodes. The capacitive performances were evaluated by the means of galvanostatic charge–discharge measurement with a window from −1 to 0 V. The capacitance was calculated from the galvanostatic discharge process according to the following equation:

(1) $$C=\frac{I\cdot \Delta t}{\Delta V\cdot m}$$

where *I* is the discharge current (A), Δ*t* is the discharge time (s), Δ*V* is the voltage difference in discharge, and *m* is the mass of the active material (g).

For the two-electrode cell, the capacitance was calculated by the Equation (2):

(2) $$C_{\mathrm{cell}}=\frac{I\cdot \Delta t}{\Delta V\cdot m_{\mathrm{total}}}$$

where m_total_ (g) is the total mass of the active material on the two electrodes.

## 3. Results and Discussion

### 3.1. Textural Characterization

**Figure 1:**
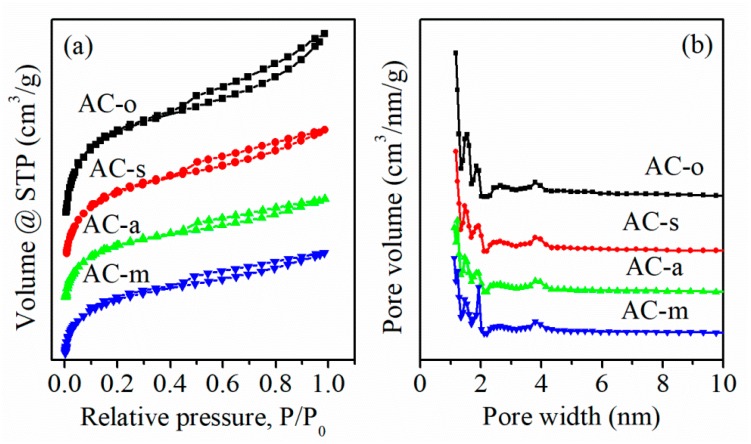
N_2_ adsorption/desorption isotherms (**a**) and pore size distributions of the samples using DFT model (**b**).

Textural properties were analyzed using N_2_ adsorption/desorption isotherms. As shown in Figure 1a, all samples displayed a notable uptake at low P/P_0_ values, and a hysteresis loop at high relative pressures, which represented the type-Ⅰ curve linked with the micropores, and the type-Ⅳ curve related to the small amount of the mesopores [11,12], respectively. On oxidation, the specific surface area was obviously reduced from 901.4 m^2^/g for AC-o, to 708.7 m^2^/g for AC-s, 490.0 m^2^/g for AC-a, and 489.3 m^2^/g for AC-m (Table 1). This evolution may arise from blocking of some pores by oxygen functional groups, destruction of pore walls, or/and the carbon shrinkage during the treatment process [13,14,15]. The pore size distribution was mainly dominated by micropores, along with a small amount of mesopores for all the samples (Figure 1b).

**Figure 2:**
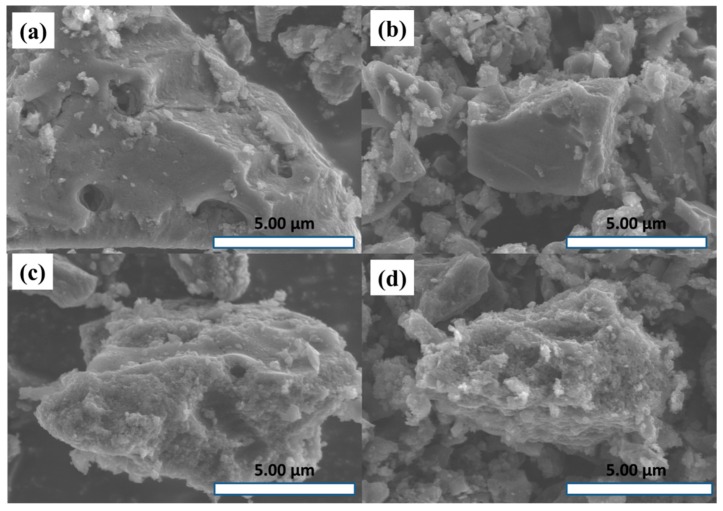
SEM images of (**a**) AC-o, (**b**) AC-s, (**c**) AC-a, and (**d**) AC-m.

Figure 2 presented SEM images of the samples. As shown in Figure 2, compared with the sample AC-o, the surface of the activated carbons after oxidation treatment was disrupted, especially the sample AC-m.

### 3.2. Surface Chemistry Property

**Figure 3:**
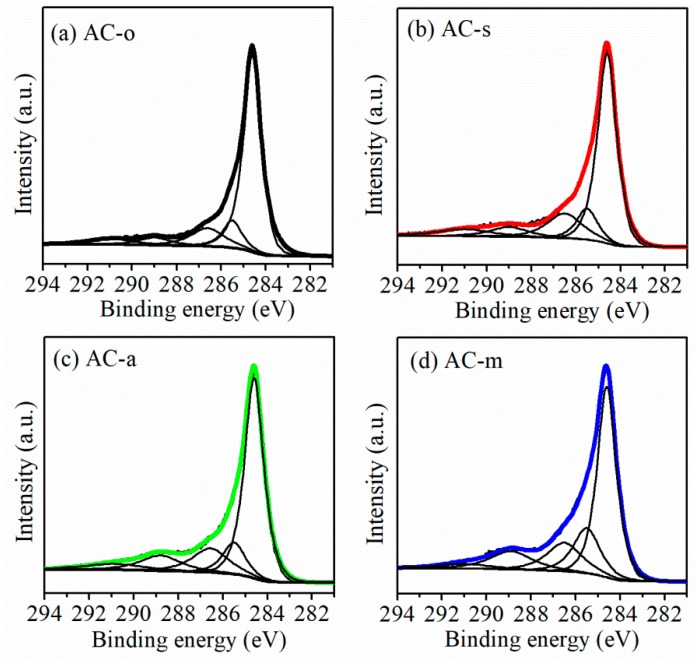
C1s XPS spectra of the samples. (**a**) AC-o, (**b**) AC-s, (**c**) AC-a, and (**d**) AC-m..

The surface chemistry property was characterized using X-ray photoelectron spectroscopy (XPS) and Boehm titration. In the XPS data (Figure 3), the increased signal at about 288.8 eV (C1s) for the treated samples, especially the samples AC-a and Ac-m, which clearly manifested the existence of sufficient carbon–oxygen bonds (O–C=O) [16,17,18] after oxidation treatment. The total oxygen amounts (Table 2) followed an upward tendency from 4.9% for Ac-o, to 6.4% for AC-s, 11.8% for AC-a, and 13.3% for AC-m. Boehm titration enabled the quantitative evaluation of oxygen functional groups (Table 3). Contents of acidic oxygen groups were extremely small on AC-o, but significantly increased upon exposure to oxidation, synchronously with the fading of the basic oxygen groups. Note that oxidation treatment, in particular using mixed oxidants, seemed to favor the generation of carboxyl groups. Combining all data above, treatment with common oxidants was clearly evidenced to be an efficient means to tune the cheap commercial ACs surface properties, especially the surface oxygen functionality. Therefore, based on four samples as electrode materials, AC-o, AC-s, AC-a, and AC-m appeared to be a good model group to assess how oxygen functionalities impact capacitive behaviors in the following.

### 3.3. Electrochemical Performance

**Figure 4:**
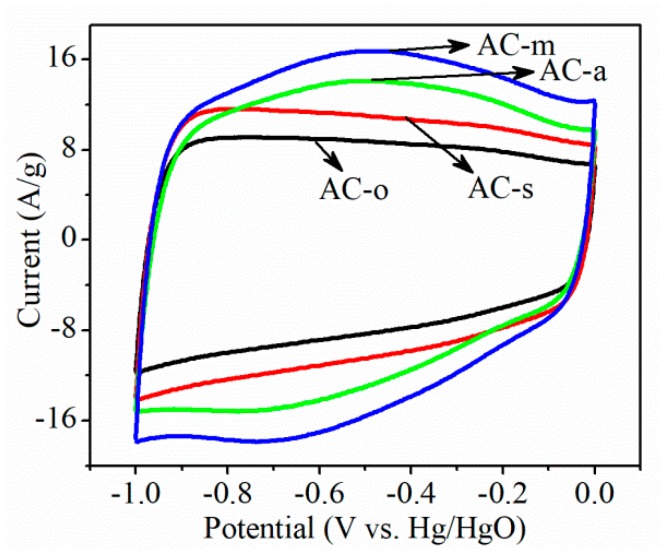
Cyclic voltammetry (CV) curves of the samples at the scan rate of 100 mV/s.

**Figure 5:**
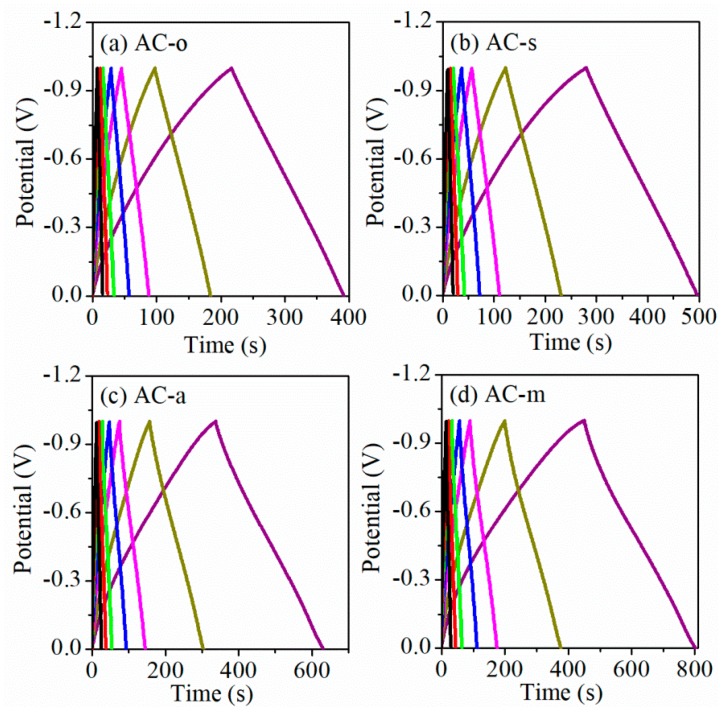
Charge-discharge curves of the samples at various current densities, from 0.5 to 10 A/g. (**a**) AC-o, (**b**) AC-s, (**c**) AC-a, and (**d**) AC-m.

**Figure 6:**
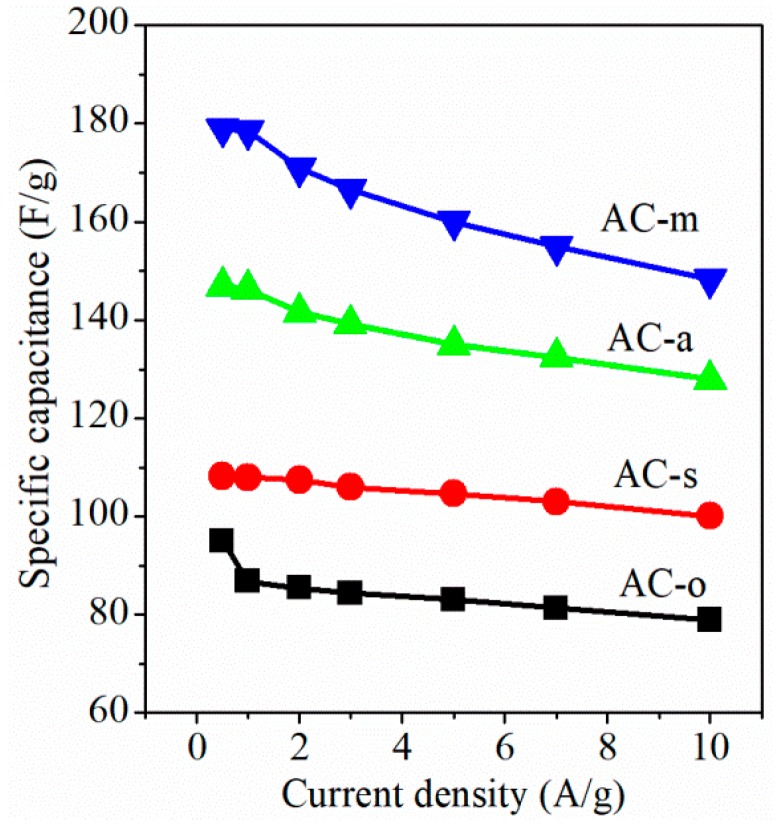
Specific capacitance as a function of applied current load.

Surface redox reaction on oxygen-functionalized AC electrodes in 6 M KOH electrolyte, using cyclic voltammetry (CV), was studied. As shown in Figure 4, the rectangular CV curves typical of electrochemical double layer capacitors were observed on AC-o and AC-s electrodes, whereas the considerably higher current response with the broad redox peak, in the potential range from −0.9 to −0.2 V over AC-a and AC-m electrodes, indicated that the salient redox reaction occurred to the AC-a and AC-m electrodes. This current response trend coincided with the oxygen content in all samples. Figure 5 and Figure 6 depicted the galvanostatic charge–discharge test at the current densities range of 0.5–10 A/g, and the corresponding specific capacitance, respectively. On the whole, electrochemical activity increased in the following order: AC-o < AC-s < AC-a < AC-m, consistent with the variation trend in oxygen contents. Particularly, taking the electrodes tested at the current load of 0.5 A/g, for example, when normalized to the weight of active materials in the electrode, specific capacitance was as low as 95.0 F/g on AC-o, but notably increased to 108.1 F/g on AC-s, 146.9 F/g on AC-a, and 179.0 F/g on AC-m. The fact that AC-o, even with much larger surface area but lower oxygen content, had much lower specific capacitance than the treated samples, tells of the importance of oxygen functional groups.

**Figure 7:**
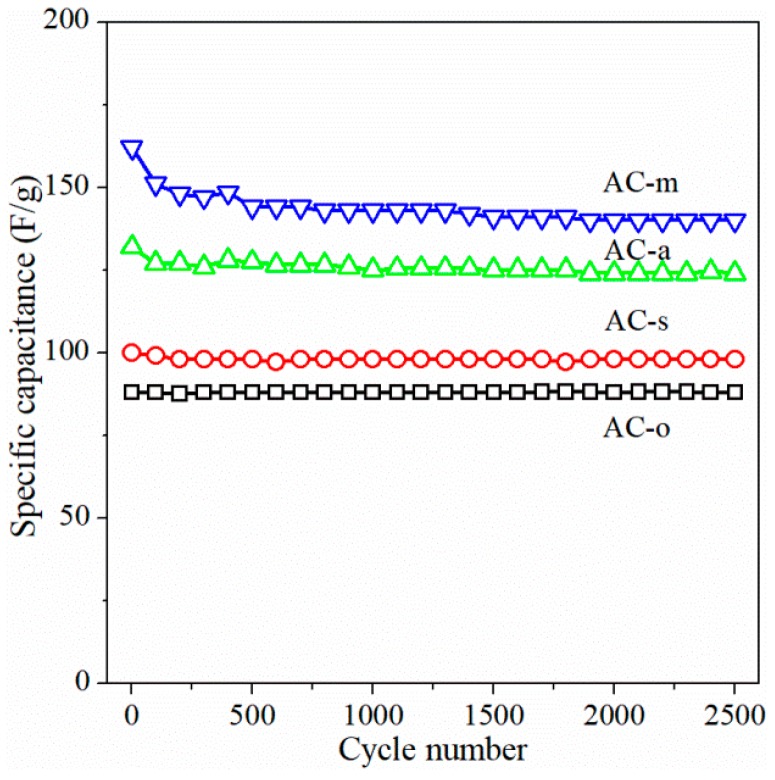
Cycling performance of the samples at a current density of 5 A/g.

Cycling stability of activated carbon electrodes was tested using constant-current charge–discharge method at a current load of 5 A/g, and the results were shown in Figure 7. The specific capacitance decreased slightly in the early 500 cycles, and was then stabilized with 86.4% retention for AC-m after 2500 cycles. By comparing the cycling performance of the four sample electrodes, the introduction of oxygen functional groups resulted in a decrease in cycle stability.

**Figure 8:**
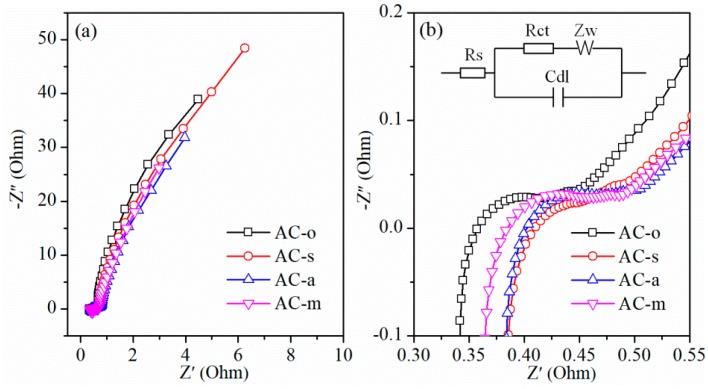
Nyquist plots (**a**) and Nyquist plots at relatively high frequency regions (**b**) of the samples. The inset showed the equivalent electrical circuit.

EIS characterization was measured with an amplitude of 5 mV in the frequency range from 10^−2^ Hz to 10^5^ Hz (Figure 8). The charge transfer resistance, fitted by ZView software using the equivalent circuit in Figure 8b, was 0.040 Ohm for AC-o, 0.023 Ohm for AC-s, 0.047 Ohm for AC-a, and 0.055 Ohm for AC-m, respectively. This result indicated that the introduction of a small number of oxygen-containing functional groups reduced charge transfer resistance, while the addition of a large number of oxygen-containing functional groups increased charge transfer resistance.

**Figure 9:**
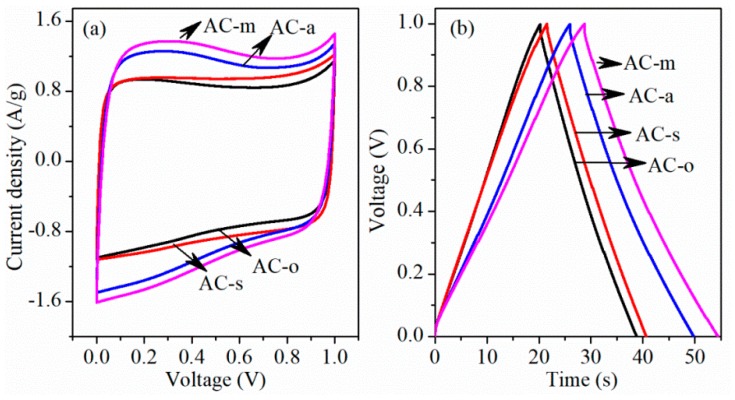
CV curves at a scan rate of 50 mV/s in 6 M KOH aqueous solution (**a**) and galvanostatic charge-discharge curves at the current density of 1 A/g (**b**) in two-electrode system.

**Figure 10:**
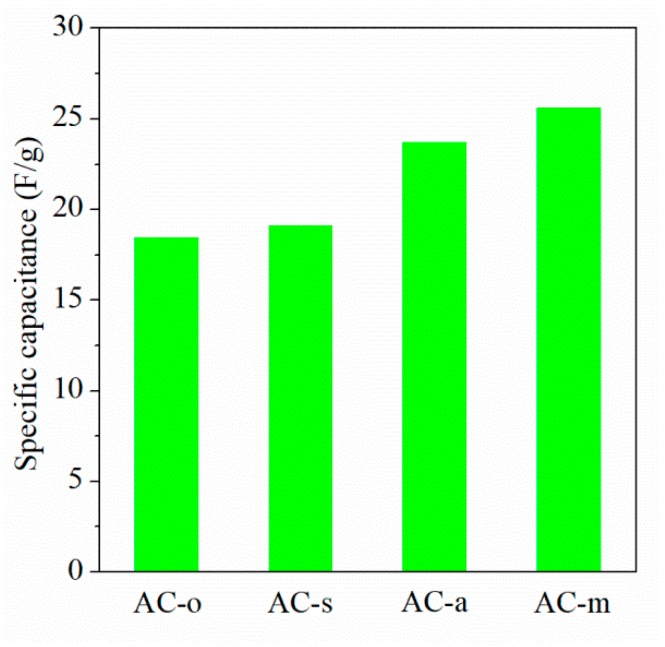
Specific capacitances of the samples in two-electrode system with the current load of 1 A/g.

To further estimate the activated carbons for the supercapacitor application, aqueous symmetrical supercapacitors were fabricated with 6 M KOH aqueous electrolytes. Figure 9a presented CV curves for a two-electrode system in the working voltage range of 0~1.0 V at a scan rate of 50 mV/s. Figure 9b displayed galvanostatic charge-discharge curves for two-electrode system at the current density of 1 A/g. According to Equation (2), the specific capacitances were 18.5 F/g for AC-o, 19.2 F/g for AC-s, 23.8 F/g for AC-a, and 25.7 F/g for AC-m, respectively (shown in Figure 10).

### 3.4. Energy Storage Mechanism Discussion

Clearly, the obviously enhanced capacitive behavior of the treated sample shown on the CV curves (Figure 4) was an evident consequence of pseudocapacitance in the KOH electrolytes. As is well known, power energy can be stored in carbon-based materials via the well-defined electric double layer mechanism, as observed here on AC-o. However, the pronounced redox peaks in CV curves clearly manifested that a new energy storage mechanism had occurred on AC-a and AC-m. This new mechanism was closely linked to the surface redox reactions, thus introducing the pseudocapacitance.

Frackowiak and coworker [18] have proposed the following surface reaction of oxygen functional groups in the aqueous medium,

(3) $$>C=O+e^{-}↔\begin{array}{cc} >C-O^{-} & \end{array}$$

which involved the reversible transformation between carbon–oxygen double bond and carbon–oxygen singer bond (where > C represented the carbon network). Meanwhile, the energy storage mechanism of oxygen functional groups was also proposed by other researchers [19,20,21]. In our case, carbon–oxygen double bonds were abundant on the AC-a and AC-m samples, owing to incorporation of carboxyl and lactone by oxidation treatment, as shown in XPS and Boehm titration results.

**Figure 11:**
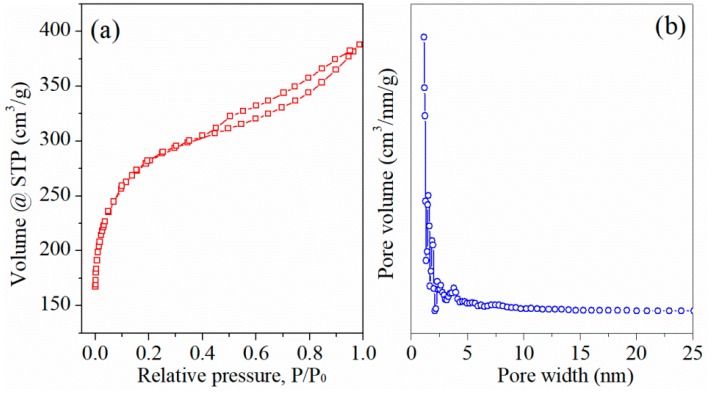
N_2_ adsorption/desorption isotherms (**a**) and pore size distribution using DFT model (**b**) of the reduced AC-m.

**Figure 12:**
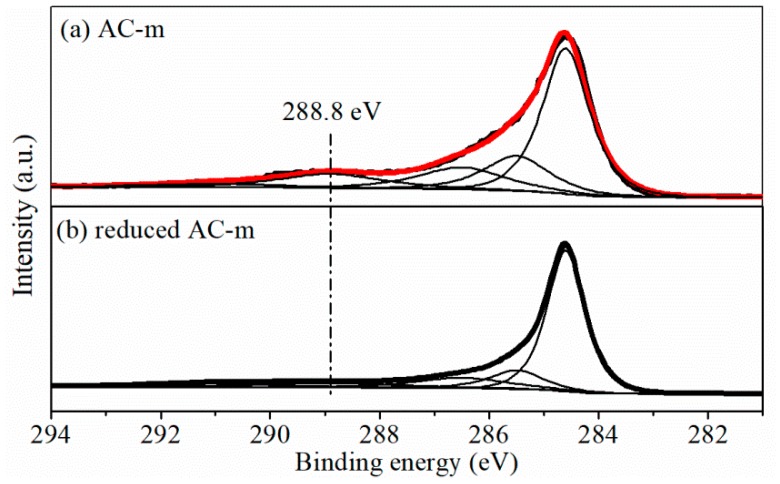
XPS C1s spectra of AC-m sample before (**a**) and after (**b**) reduced thermal treatment in 4% H_2_ and 96% Ar at 900 °C for 3 h.

**Figure 13:**
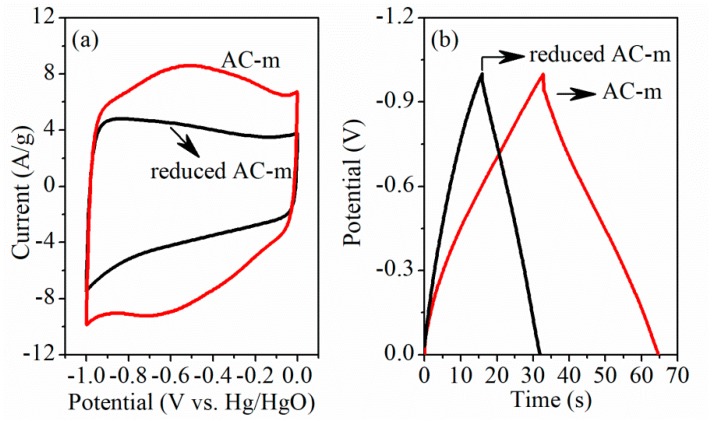
CV curves of AC-m before and after thermal reduction at the scan rate of 50 mV/s (**a**) and constant-current charge–discharge test with the current load of 5 A/g (**b**).

Contribution of oxygen functional groups to specific capacitance in ACs was further evidenced by comparing electrochemical performance of AC-m before and after reduction treatment (in 4% H_2_ and 96% Ar at 900 °C for 3 h). Surprisingly, N_2_ adsorption/desorption test (Figure 11) displayed that the specific surface area and average pore diameter of reduced AC-m obviously was 907.5 m^2^/g and 2.64 nm, similar to those of the AC-o sample (901.4 m^2^/g and 2.65 nm). XPS analysis (Figure 12) showed that the reduced heat treatment decreased the contents of oxygen functional groups on the AC surface. The intensities of the distinct C1s peaks, assigned to carboxyl group at approximately 288.8 eV [22], was greatly reduced relative to those of sp^3^ (285.5 eV) and sp^2^ (284.6 eV) hybridized carbon [23] when exposed to thermal treatment, and the corresponding electrochemical performance were shown in Figure 13. The redox signals disappeared, and current response and specific capacitance decreased considerably upon thermal treatment to reduce oxygen functional groups. For example, the specific capacitance of AC-m decreased from 158.0 F/g to 79.8 F/g at a current load of 5 A/g upon thermal reduction. These outcomes further confirmed that the improving electrochemical behavior was responsible for redox reaction of oxygen functional groups.

The energy storage mechanism helped to understand the superficial reaction for the excellent electrochemical capacitive performance of the treated samples. Likewise, the other mechanisms, e.g., the double layer energy storage mechanism, might contribute to the observed capacitor performance, especially porous carbon materials, in that the reduced AC-m electrode also delivered a certain capacitance. Therefore, the outstanding capacitive performance of oxygen–carbon was attributed to the double-modal energy storage mechanisms: (1) the adsorption/desorption of the active ions in the electric double layer; (2) reversible surface reaction of oxygen functionality. Furthermore, the improved surface wettability of oxygen-functionalized carbon materials contributed electrolyte effectively into the pores of carbon materials, and formed an electric double layer between active ions and inner pore wall.

## 4. Conclusions

Carbon electrode materials for double layer capacitors have attracted much attention, due to the low cost and abundant resources. However, the low specific capacitance hindered the development of carbon electrode materials. This paper reported a class of oxygen-functionalized carbon electrodes for supercapacitors. The oxygen-functionalized carbon materials can be facilely derived by using a simple, operable, reproducible oxidation treatment of the cheap commercial ACs, and show excellent capacitive performance, which could be attributed to the pseudocapacitance of the redox reaction of oxygen functional groups. The low cost and abundant resources render the oxygen-functionalized carbon materials as a promising candidate for practical use.

## Figures and Tables

**Table 1:** Structural parameters calculated from N_2_ adsorption/desorption isotherms.

Sample	S_BET_ (m^2^/g)	V_mic_ (cm^3^/g)	V_meso_ (cm^3^/g)	V_t_ (cm^3^/g)	Average Pore Diameter (nm)
AC-o	901.4	0.373	0.163	0.596	2.65
AC-s	708.7	0.290	0.109	0.443	2.50
AC-a	490.8	0.196	0.084	0.311	2.53
AC-m	489.3	0.200	0.078	0.310	2.54

**Table 2:** Carbon and oxygen contents in the studied samples obtained from XPS.

Samples	C (%)	N (%)	O (%)	O/C (%)
AC-o	94.41	0.65	4.94	5.23
AC-s	93.11	0.54	6.35	6.82
AC-a	87.55	0.69	11.76	13.43
AC-m	85.99	0.74	13.28	15.44

**Table 3:** Contents of oxygen functional groups in the studied samples obtained from Boehm titration.

Sample	Carboxyl (mmol/g)	Lactone (mmol/g)	Phenol (mmol/g)	Total Acidic Groups (mmol/g)	Total Basic Groups (mmol/g)
AC-o	0.103	0.099	0.161	0.431	0.292
AC-s	0.602	0.102	0.081	0.940	0.059
AC-a	1.967	0.256	0.028	2.929	-
AC-m	2.416	0.324	0.158	3.526	-
